# Human sporadic breast carcinoma histotypes driven by the Human Betaretrovirus homologous to Mouse Mammary Tumor Virus

**DOI:** 10.1002/ijc.35438

**Published:** 2025-04-11

**Authors:** Prospero Civita, Chiara Maria Mazzanti, Francesca Lessi, Caterina Marchiò, Cristian Scatena, Michele Menicagli, Matteo Ghilli, Manuela Roncella, Antonio Giuseppe Naccarato, Anna Sapino, Jacob Hochman, Mauro Pistello, Generoso Bevilacqua

**Affiliations:** ^1^ Fondazione Pisana per la Scienza San Giuliano Terme Pisa Italy; ^2^ Department of Medical Science University of Turin Turin Italy; ^3^ Candiolo Cancer Institute, FPO‐IRCCS Candiolo Turin Italy; ^4^ Department of Translational Research and New Technologies in Medicine University of Pisa Pisa Italy; ^5^ Breast Cancer Center Pisa University Hospital Pisa Italy; ^6^ Alexander Silberman Institute of Life Sciences The Hebrew University of Jerusalem Jerusalem Israel; ^7^ Present address: School of Pharmacy and Pharmaceutical Sciences College of Biomedical and Life Science, Cardiff University Cardiff UK

**Keywords:** breast cancer etiology, breast cancer histotype, human betaretrovirus‐HBRV, human mammary tumor virus‐HMTV, mouse mammary tumor virus‐MMTV

## Abstract

The viral hypothesis for human sporadic breast carcinoma is based on the murine model of Mouse Mammary Tumor Virus (MMTV)‐induced mammary tumors. Known risk factors like estrogens, obesity, and alcohol do not play a direct causal role. The Human Betaretrovirus (HBRV), also called Human Mammary Tumor Virus (HMTV), is the human homolog of MMTV, implicated in sporadic breast carcinoma (80% of ductal carcinoma in situ and 40% of invasive tumors). In contrast, hereditary breast carcinomas lack viral sequences. Murine mammary tumor histotypes are determined by specific viral strains activating definite molecular pathways via insertional mutagenesis. Similarly, the diverse histotypes observed in human invasive breast carcinoma may be influenced by a viral etiology. A study of 253 invasive breast carcinoma cases, representing 15 histotypes, detected HBRV/MMTV‐*ENV* sequences in 20%, consistent with international literature. All histotypes tested positive except those linked to hereditary syndromes, such as medullary, apocrine, and metaplastic carcinoma. This distinction reinforces the reported lack of association between HBRV/HMTV and hereditary breast cancer, while supporting a viral etiology for sporadic carcinoma. Relevant characteristics of sporadic histotypes align with the “hit and run” hypothesis of viral carcinogenesis. Histotype differences may result from molecular pathways activated by Int genes, though mechanism beyond insertional mutagenesis and the possibility of specific HBRV strains cannot be ruled out. The potential for detected viral sequences to originate in human tumors from endogenous MMTV or contamination with murine material is critically examined.

## Graphical abstract

What's new?

While estrogens, obesity, and alcohol are recognized risk factors for breast cancer, a viral hypothesis has been suggested based on the murine model of Mouse Mammary Tumor Virus (MMTV)‐induced mammary tumors. This study analyzed sequences of the human homolog of MMTV across human breast carcinoma histotypes, detecting the virus in all types except hereditary‐associated ones. The findings reinforce the lack of association between HMTV/HBRV and hereditary carcinomas and support a viral etiology for sporadic breast cancer. Histotype differences may result from molecular pathways activated by Int genes, although mechanisms beyond insertional mutagenesis and distinct HBRV strains cannot be ruled out.

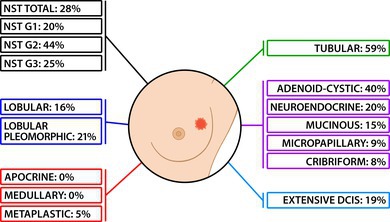

**Abbreviations**
ADH: Atypical Ductal Hyperplasia
AKT: AK Strain Transforming, Protein Kinase B
BC: Breast Cancer
BRCA: Breast Cancer gene
CIS: Common Integration Site
CISH: Chromogenic In Situ Hybridization
CK2: Casein Kinase 2
DCIS: Ductal Carcinoma In Situ
DNA: Deoxyribonucleic Acid
E2F: Early Region 2 Binding Factor, transcription factor
EBV: Epstein–Barr Virus
EGFR: Epidermal Growth Factor Receptor
EMT: Epithelial Mesenchymal Transition
ENV: Envelope
ER: Estrogen Receptor
ERV: Endogenous Retrovirus
FFPE: Formalin‐Fixed Paraffin‐Embedded
FGF: Fibroblast Growth Factor
GAG: Group Specific Antigen
GEMM: Genetically Engineered Mouse Model
GSK3: Glycogen Synthase Kinase
HAN: Hyperplastic Alveolar Nodule
HBRV: Human Betaretrovirus
HBV: Hepatitis B Virus
HCV: Hepatitis C Virus
HER2: Human Epidermal Growth Factor Receptor 2
HERB2: Erythroblastic Oncogene B, receptor tyrosine‐protein kinase erbB‐2
HERV: Human Endogenous Retrovirus
Hippo: Salvador‐Warts‐Hippo (SWH) pathway
HIV: Human Immunodeficiency Virus
HML: Human MMTV‐Like
HMTV: Human Mammary Tumor Virus
HPV: Human Papilloma Virus
H‐RAS: Harvey Rat Sarcoma Virus gene
IAP: Intracisternal type A Particle
IARC: International Agency for Research on Cancer
IHC: Immunohistochemistry
Int: Integration locus or gene
ITAM: Immunoreceptor Tyrosine‐based Activation Motif
JSRV: Jaagsiekte Sheep Retrovirus
K‐RAS: Kirsten Rat Sarcoma Virus gene
KSHV: Kaposi's Sarcoma‐associated Herpesvirus
L: Low Incidence
LTR: Long Terminal Repeat
MET: from the mutagenic compound N‐METhyl‐N'‐nitro‐N‐nitrosoguanidine, oncogene with tyrosine kinase activity
miRNA: microRNA
MMTV: Mouse Mammary Tumor Virus
mTOR: Mammalian or Mechanistic Target of Rapamycin
Mtv: Endogenous MMTV
Muc‐PyMT10C: Mucin‐Polyoma Middle T, lentivirus
MYC: MYeloCytomatosis, family of regulator genes and proto‐oncogenes
NAF: Negative Acting Factor
NST: No Special Type
OPA: Ovine Pulmonary Adenocarcinoma
P: Plaque
PBC: Primary Biliary Cholangitis
PCR: Polymerase Chain Reaction
PI3K: Phosphoinositide 3‐Kinase
PKC: Protein Kinase C
PR: Progesterone Receptor
PTEN: Phosphatase and Tensin homolog
PyMT: Polyoma Middle T
Rap1: Ras‐proximate‐1
RB: Retinoblastoma gene
REM: Regulator of Expression/Export of MMTV mRNA
RNA: Ribonucleic Acid
RT‐PCR: Real‐Time PCR
S: Standard
SAG: Superantigen
SLC12A7: Solute Carrier family 12, member 7
SP: Signal Peptide
SRC: Tyrosine‐Protein Kinase, SaRComa
SU: Surface Unit
U3: Unique, 3′ end
VEGF: Vascular Endothelial Growth Factor
WAP: Whey Acidic Proteins
WHO: World Health Organization
Wnt: Wingless‐related integration Site or Wingless and Int‐1

## 1 INTRODUCTION

Current data suggest that a viral agent is the most likely etiological factor for sporadic breast cancer (BC).^1^, ^2^ While estrogens, obesity, and alcohol are recognized as significant risk factors, their direct causative roles have not been demonstrated. These factors promote cell proliferation, with estrogens acting directly, adipose tissue contributing through the synthesis of endogenous estrogens, and alcohol stimulating endogenous estrogen synthesis.^3^, ^4^, ^5^ Thus, they are better categorized as promoters rather than initiators of cancer. The International Agency for Research on Cancer (IARC) monograph on the carcinogenic risk of alcohol, published in 2010 and still frequently cited, concludes that there is a “*sufficient* evidence in humans for the carcinogenicity of alcoholic beverages”. Nonetheless, it also asserts that “the occurrence of malignant tumors of … and breast is *causally* related to the consumption of alcoholic beverages”. However, the IARC's conclusions, primarily based on epidemiological data, have certain limitations: (a) in nearly all cited experimental studies, alcohol was combined with potent carcinogens, underscoring its role as a promoter rather than an initiator; and (b) the role of oncogenic viruses—such as HPV in the oropharynx, EBV in the esophagus and stomach, and HBV and HCV in the liver—is either superficially addressed or entirely overlooked. Moreover, other studies have failed to demonstrate a link between alcohol consumption and BC.^6^

The viral hypothesis for BC is based on the experimental model of murine mammary tumors induced by the Mouse Mammary Tumor Virus (MMTV), a non‐acute, slow‐transforming betaretrovirus transmitted to offspring through maternal milk.^2^ MMTV is a potent oncogenic virus that exerts its transforming effect primarily through insertional mutagenesis, integrating into specific gene locations known as Int (integration) loci. These loci host genes involved in tissue growth and development, including *WNT1* (Int1), *FGF3* (Int2), and *NOTC4* (Int3), among others.^7^

The activation of Int genes is directly responsible for the development of preinvasive/preneoplastic lesions, such as typical and atypical hyperplasia and carcinoma in situ, under the indispensable proliferative stimulus of estrogens. On the other hand, disease progression—leading to invasive carcinoma and subsequent metastatic spread—is likely driven by specific molecular pathways (Wnt/β‐catenin, FgF, Notch, and others), which are, in turn, activated by Int genes.^7^

Unlike other retroviruses, which exhibit some preference for specific integration sites, MMTV inserts randomly, demonstrating the most random distribution of integration sites observed to date.^8^ As a result, large amounts of virus must be produced, and many mammary cells must be infected for tumorigenesis to occur. The randomness of insertion, along with the complexity of the molecular events involved, accounts for the long latency period of MMTV‐induced tumors, which can sometimes last up to 1 year.^9^

Variants of the MMTV provirus with specific deletions in the U3 region of the LTR have been shown to play an etiological role in murine T‐cell lymphomas.^10^, ^11^ The close interactions^2^ that MMTV establishes with the immune system during the host infection likely facilitate the development of this type of neoplasia.

Diverse MMTV strains have been identified and are named after the mouse strain hosting them, such as MMTV‐C3H and MMTV‐RIII, or designated by letters, such as S (standard, the classic Bittner virus), P (plaque), L (low incidence), among others.^12^

The MMTV model has been crucial in advancing our understanding of human BC biology, contributing to concepts such as tumor progression, preneoplastic/preinvasive lesions, and hormone dependence.^7^ The potential role of MMTV in BC etiology has been hypothesized since the 1970s. In 1972 Axel, Gulati, and Spiegelman identified viral particles containing RNA polymerase and viral RNA in up to 80% of human breast carcinomas cases.^13^ In 1995, Beatriz Pogo and colleagues detected MMTV‐like sequences in invasive human BC cases at a notable frequency of nearly 40%^14^ and later successfully identified and amplified the proviral structure.^15^ A 2006 study utilizing laser microdissection of tumor cells and fluorescent nested PCR reported a positivity rate of 33%, further strengthening the evidence.^16^ A recent review of 46 studies involving 51 patient cohorts found that viral sequences were detected in 42 cohorts worldwide, comprising 5015 cases, with 1320 testing positive. The overall positivity rate was 26%.^2^

Intriguingly, viral sequences have been detected in normal epithelial cells adjacent to invasive carcinoma (~20%) and in preinvasive breast lesions. Among these lesions, 27% of atypical ductal hyperplasia (ADH) cases and, surprisingly, a striking 82% of ductal carcinoma in situ (DCIS) cases exhibited the presence of the virus.^17^ The very high percentage of positive DCIS cases is halved during the transition to the invasive form, accompanied by a strong signal reduction observed with CISH (chromogenic in situ hybridization) and a dramatic decrease in viral sequence copy number, as demonstrated by quantitative PCR.^17^

Notably, viral sequences have even been detected in non‐cancerous breast biopsies taken 1 to 11 years before cancer development.^18^ MMTV sequences have also been identified in hormone‐influenced human tumors, such as those of the ovary, endometrium, and prostate.^19^

Interestingly, MMTV‐like sequences have been detected in human saliva.^20^ Remarkably, saliva samples from BC patients showed a detection rate of nearly 60%. In contrast, saliva samples from healthy adults tested positive in 11% of cases, while normal salivary glands were positive in 8% of cases.

Importantly, hereditary BRCA‐related breast cancers have tested negative for MMTV‐like sequences.^21^ Hereditary BCs have a distinct etiopathogenesis, driven primarily by inherited gene alterations in germline cells. As a result, external factors, such as viruses, are likely not required for their development.

The MMTV proviral structure has been identified in human breast cancer cells and found integrated into the DNA of cancer cells,^15^, ^22^ while infectious MMTV particles have been isolated from breast cancer cells.^22^, ^23^

Of particular relevance is the finding that MMTV can infect human mammary cells, as demonstrated by Indik and colleagues.^8^, ^22^, ^24^ The infected cells can spread the infection to other cells, eventually involving the entire cell population. Stimulation of these cells with dexamethasone induced the synthesis of viral structural proteins and the production of mature, infectious type B particles. Notably, in these infected human cells, MMTV integration occurred randomly.^8^

Preliminary data from Andrew Mason's laboratory demonstrate HBRV insertion in human breast carcinomas, with integration proximal to cancer‐related genes such as *SLC12A7*.^25^

MMTV is linked not only to BC but also to primary biliary cholangitis (PBC), an autoimmune liver disease.^26^ In this context, the proviral genome has been identified, viral B particles have been isolated, and more than 3400 integration sites have been detected. Notably, biliary epithelial cells have also been successfully infected with the virus.^26^

Based on the murine model, human infection with MMTV has been hypothesized to occur either through breastfeeding or direct transmission from mice. However, both hypotheses have significant limitations:

1. Human milk typically contains negligible or no MMTV particles. Moreover, contemporary breastfeeding practices are relatively short in duration, whereas the murine model suggests that infection requires large quantities of the virus and prolonged breastfeeding. This extended duration compensates for the destruction of most viral particles by gastric juices. Additionally, human milk has been shown to have a destructive effect on MMTV.^2^
2. Direct transmission from mice to humans also appears unlikely, as it would require prolonged and close cohabitation, which is uncommon in typical human–mouse interactions.

The existence of a human viral counterpart to MMTV has long been hypothesized, leading to the introduction of the term Human Mammary Tumor Virus (HMTV) in 2001.^15^ Recently, the existence of a human Betaretrovirus homologous to MMTV was confirmed through the detection of viral sequences highly similar to MMTV in the remains of Copper Age individuals, approximately 4500 years old.^27^ It is highly plausible that a cross‐species transmission event occurred around 10,000 years ago, coinciding with the advent of agriculture in the *Fertile Crescent* region. This environmental shift facilitated the coexistence of humans, mice, and other animals, leading to the transmission of various animal pathogens to humans. Subsequently, saliva may have emerged as a route for human‐to‐human transmission. In 2004, Andrew Mason introduced the term Human Betaretrovirus (HBRV).^28^ This renaming was based on two key considerations: first, the human virus has diverged from its direct link to mice, warranting a distinct designation; second, HBRV has been associated with diseases beyond breast pathology. Notably, HBRV remains the only known betaretrovirus in humans.

The microscopic architecture of murine mammary tumors was first described in 1958 by Thelma Brumfield Dunn, who identifies two main types of neoplasia.^29^ Type A tumors exhibited “a fine uniform acinar structure”, while type B tumors “represented a diversified, multiform group in which the tumor is clearly of glandular origin, but in which there are no predominant features”. Type B tumors were characterized by a mixture of acinar structures, irregular glands, papillary projections, and other architectural elements.

Sixty years ago, Francesco Squartini^30^ demonstrated that the biological and morphological diversity of mammary tumors across different mouse strains depended on the specific MMTV strain harbored, rather than the genetic background of the animals. For instance, MMTV‐C3H induces hormone‐independent, highly metastatic type B adenocarcinoma, with HAN (hyperplastic alveolar nodules) as preneoplastic lesions. In contrast, MMTV‐RIII induces hormone‐dependent, non‐metastatic type A adenocarcinoma, with plaques as preneoplastic lesions.

Human breast carcinoma exhibits a variety of histological architectures, which can be attributed to the molecular characteristics of the neoplasm, as detailed in the Discussion section. However, no etiological correlation has been identified to date.

Given insights from the MMTV murine model and the frequent detection of HBRV sequences in human BC, we aimed to investigate the distribution of viral positivity across different BC histotypes. This study explores a potential correlation between the viral agent and tumor architecture.

## 2 MATERIALS AND METHODS

### 2.1 specimens

Formalin‐fixed, paraffin‐embedded (FFPE) tissue samples of invasive breast carcinoma were obtained from the pathology archives of the Universities of Pisa and Turin. Diagnoses were established accordingly to the 2019 WHO Classification of Tumors, distinguishing between “no special type—NST” and “special type” carcinomas. NST tumors, which comprise 75%–80% of cases, lack distinctive histological features and are graded G1–G3 based on mitotic count, nuclear atypia, and tubule formation. The remaining 20%–25% of cases consist of histologically distinct subtypes, with lobular carcinoma accounting for 10%, while other types are rare.

**FIGURE 1:**
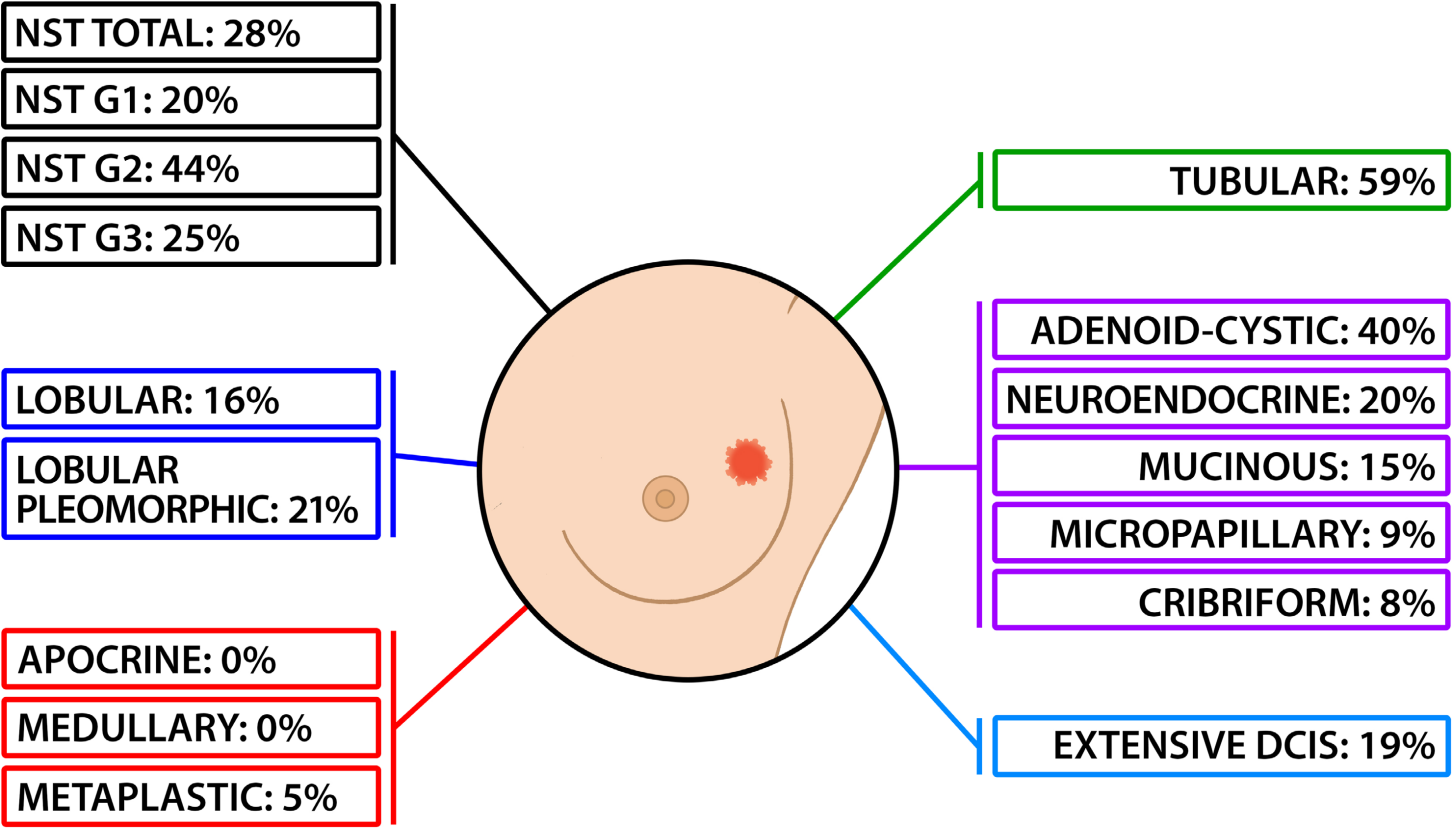
Graphic representation of HBRV distribution among human breast carcinoma histotypes. It is worth noting: (A) tubular carcinoma exhibits a very high positivity rate (59%), likely due to its high degree of differentiation. (B) Apocrine, medullary, and metaplastic carcinomas, all associated with hereditary cancer syndromes, show absent or nearly absent positivity.

The study analyzed 253 cases of BC (Table 1 and Figure 1), including 227 invasive tumors of various histotypes and 26 cases of extensive ductal carcinoma in situ. Among NST tumors, 58 cases were identified, including 5 G1, 9 G2, and 44 G3. Special type carcinomas (169 cases) included adenoid cystic, apocrine, cribriform, lobular, lobular pleomorphic, medullary, metaplastic, micropapillary, mucinous, neuroendocrine, and tubular subtypes.

**TABLE 1:** HBRV in human breast carcinoma histotypes.

Histotype	Total number	No. of positive cases	% of positivity
(A) Types positive for HBRV			
(a) Invasive tumors			
NST (no special type)	58	16	28%
○ G1	5	1	20%
○ G2	9	4	44%
○ G3	44	11	25%
Special types			
Adenoid cystic	5	2	40%
2 Cribriform	13	1	8%
3 Lobular	19	3	16%
4 Lobular pleomorphic	14	3	21%
5 Metaplastic	20	1	5%
6 Micropapillary	22	2	9%
7 Mucinous	26	4	15%
8 Neuroendocrine	5	1	20%
9 Tubular	22	13	59%
Total	204	46	22.5%
(b) DCIS associate to invasive tumors			
DCIS extensive	26	5	19%
Total (a + b)	230	49	21%
(B) Types negative for HBRV			
(a) Invasive tumors			
Special types			
Apocrine	16	0	0%
2 Medullary	7	0	0%
Total	23	0	0%
Grand total	253	51	20%

*Note*: The overall positivity rate was 20%. NST carcinomas showed a 28% of positive cases, with the G2 group exhibiting double the positivity rate of G1 and G3. Sixteen cases of apocrine carcinoma and seven cases of medullary carcinoma showed no viral sequences. Metaplastic carcinoma was largely negative. Cribriform and micropapillary carcinomas had a positivity rate of <10%, while other histotypes ranged from 15% in mucinous carcinoma to 59% in tubular carcinoma. The mean positivity rate was 22.5% for pure invasive tumors and 21% when extensive DCIS was included. Extensive DCIS exhibited a 19% positivity rate, similar to pure invasive tumors.

Relevant for the discussion: (a) tubular carcinoma is a rare histotype characterized by highly differentiated tubular structures with open lumina lined by a layer of epithelial cells; this variant has an excellent prognosis; (b) extensive DCIS refers to DCIS associated with invasive carcinoma, where the intraductal component constitutes >25% of the invasive carcinoma area and extends into surrounding breast tissue.

### 2.2 laser microdissection

Epithelial cell populations were selected for molecular analysis using a Leica automatic laser microdissector. Six‐μm sections were obtained from each case using a new microtome blade per block, ensuring the exclusion of stromal and inflammatory cells.

### 2.3 molecular analysis

#### 2.3.1 ENV analysis

Detection of MMTV/HMTV‐*ENV* sequence was performed as described by Beatriz Pogo^14^ and subsequent studies.^2^ This sequence analysis approach is highly reliable for FFPE tissues. The *ENV* gene is of particular interest due to its potential role as an oncogene,^31^ as discussed later. Fluorescence‐seminested PCR was employed to detect *ENV* DNA. Primers were designed based on GenBank sequence AF243039. Outer primers produced a 201‐bp fragment, while the inner primers yielded a 191‐bp fragment.

*Outer primers*:

- Forward: 5′‐GATGGTATGAAGCAGGATGG‐3′
- Reverse: 5′‐AAGGGTAAGTAACACAGGCAGATGTA‐3′.

*Inner primers* (semi‐nested PCR):

- Forward: 5′‐AGCAGGATGGGTAGAACCTAC‐3′
- Reverse: same as outer reverse primer.

*PCR conditions*:

- First round PCR: 50 μL reaction containing 1× standard PCR buffer, 1.5 mM MgCl₂, 200 μM dNTPs, 0.5 μM unlabeled reverse primer, 0.5 μM 6‐FAM labeled forward primer, 2.5 U AmpliTaq Gold (Applied Biosystems), and 500 ng genomic DNA.
- Second round PCR: 2 μL of first‐round PCR product in a 50 μL reaction.
- Thermal cycling: initial denaturation: 94°C for 10 min; first‐round: 40 cycles (94°C for 45 s, 58°C for 45 s, 72°C for 60 s); second‐round: 30 cycles (same as first‐round conditions); final extension: 72°C for 7 min.

**FIGURE 2:**
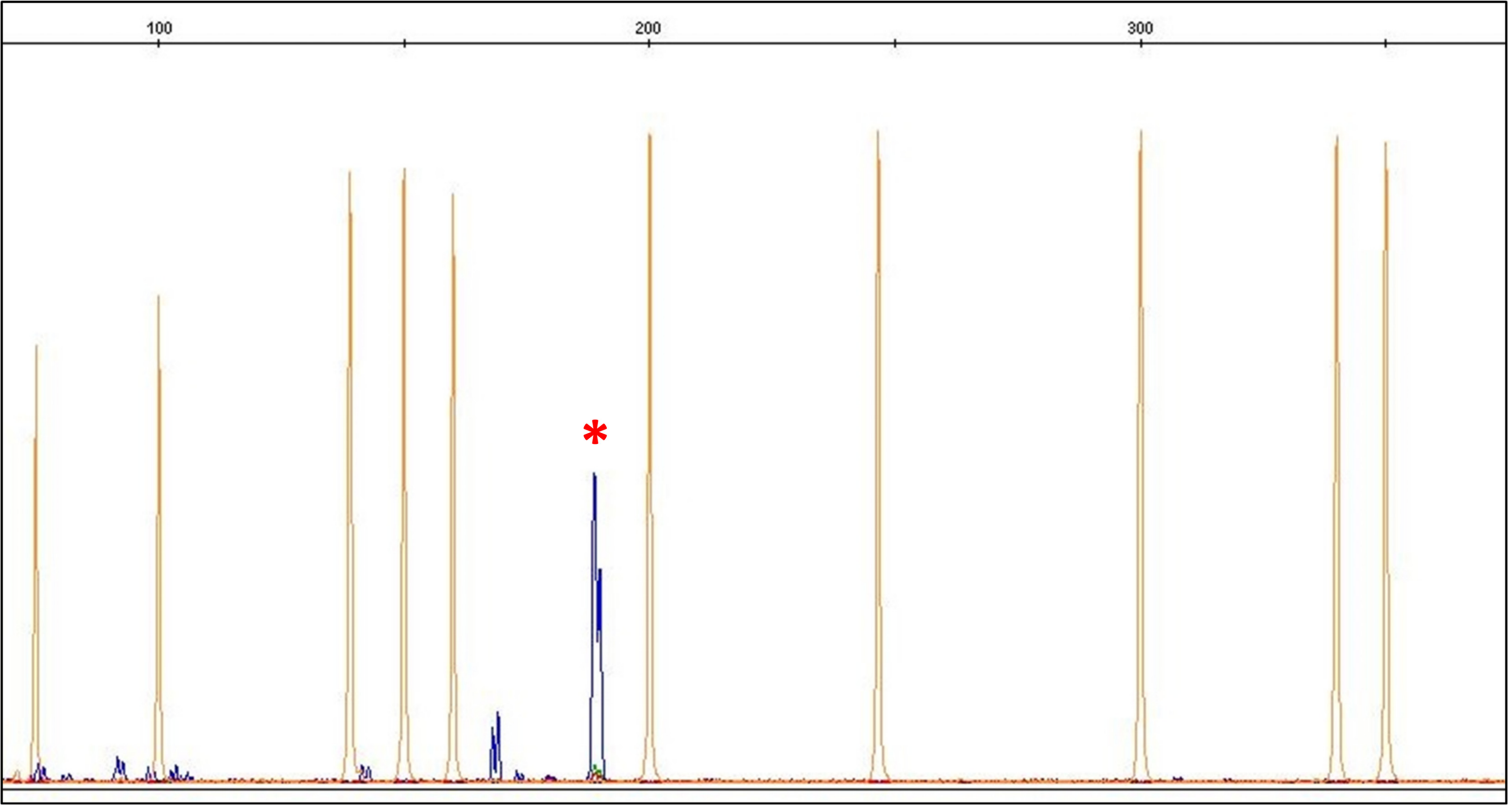
Example of PCR detection of an MMTV/HBRV *ENV* sequence positive case. Representative capillary electrophoresis image obtained via fluorescent fragment analysis, showing a positive case for *ENV* sequence amplification. The expected amplicon is 191 bp (red asterisk) after the semi‐nested PCR. Yellow peaks represent the ROX‐labeled size standard.

**FIGURE 3:**
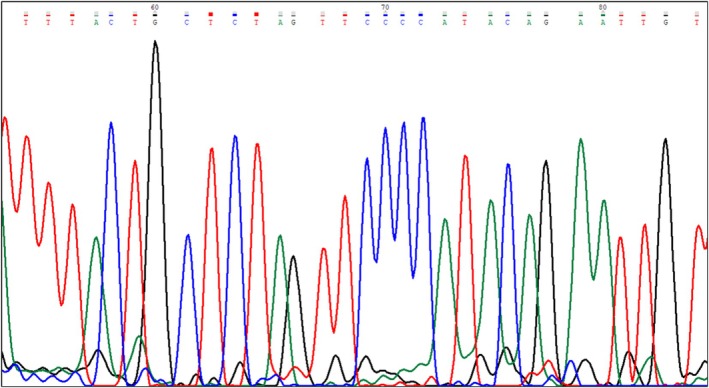
Confirmation of viral sequence by Sanger sequencing. A portion of the amplified fragment sequence is shown, confirming alignment with GenBank accession number AF243039. This validates that the amplified PCR product corresponds to the MMTV‐*ENV* region of the MMTV virus. The analysis was performed using the Sanger sequencing assay.

Fluorescent amplicons were analyzed by capillary electrophoresis. A 3 μL nested‐PCR product was mixed with 0.5 μL ROX‐labeled size standard (Gene Scan 400 HD ROX; Applied Biosystems) and 11.5 μL Hi‐Di Formamide, denatured at 95°C for 3 min, and loaded onto an ABI PRISM 3100 genetic analyzer (GENESCAN software, v3.1). The ENV PCR fragment was purified (QIAquick Gel Extraction Kit, Qiagen) and sequenced (ABI PRISM 3130XL). Sequences were aligned via BLAST (http://blast.ncbi.nlm.nih.gov) against MMTV/HMTV sequences in GenBank (Figures 2 and 3).

#### 2.3.2 Mouse DNA contamination

**FIGURE 4:**
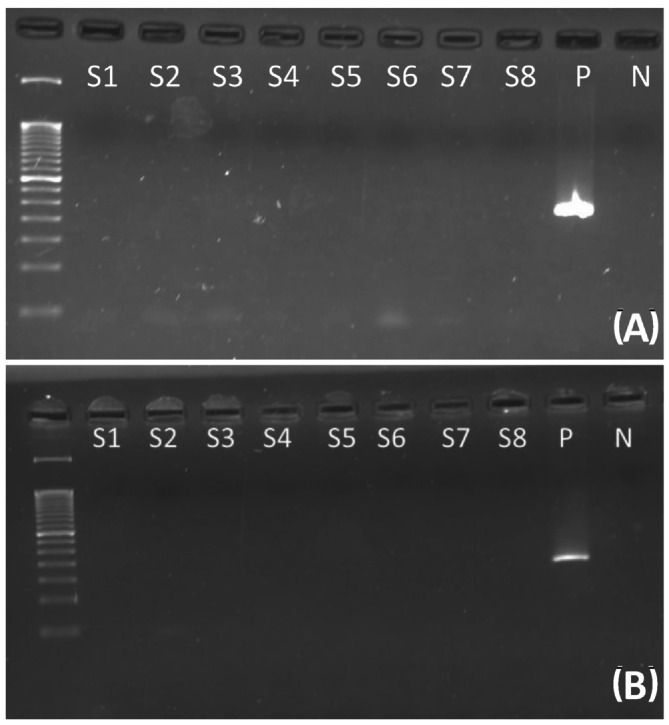
Absence of contaminating murine DNA, electrophoresis gel of murine DNA amplification. (A) Murine IAP—Intracisternal A Particles LTRs. DNA from human HBRV‐*ENV* positive cases tested negative for IAP DNA. The expected amplicon is 250 bp. S1–S8: Representative negative human cases. P: Murine positive control. N: Negative PCR control. A 50 bp DNA ladder was used as the molecular size marker. (B) Murine Mitochondrial DNA. DNA from human HBRV‐*ENV* positive cases tested negative for Murine Mitochondrial DNA. The expected amplicon is 153 bp. S1–S8: representative negative human cases. P: murine positive control. N: negative PCR control. A 50 bp DNA ladder was used as the molecular size marker.

Murine DNA contamination was ruled out by amplifying murine DNA and intracisternal A‐particle (IAP) sequences per Robinson et al.^32^ (Figure 4).

(1) *Murine Mitochondrial DNA Detection*. PCR (286 bp fragment) followed by semi‐nested PCR (153 bp fragment) was performed:

*First‐round primers*:

- Forward: 5′‐AGACGCACCTACGGTGAAGA‐3′.
- Reverse: 5′‐AGAGTTTTGGTTCACGGAACATGA‐3′.

*Semi‐nested PCR primer*:

- Forward: 5′‐TGCCAAACCCCAAAAACACT‐3′ (reverse same as first‐round).

Reactions (50 μL) contained: 1× TaqGold buffer, 1.5 mM Mg^2+^, 200 μM dNTPs, 2.5 pmol of each primer, 0.25 U TaqGold polymerase, and 3–5 μL DNA.

*Thermal cycling*: 94°C for 7 min → 30 cycles (94°C for 30 s, 58°C for 30 s, 72°C for 45 s) → final extension 72°C for 7 min.

(2) *IAP Sequence Detection*. PCR (250 bp fragment) was performed with:

- Forward primer: 5′‐ATAATCTGCGCATGAGCCAAGG‐3′.
- Reverse primer: 5′‐AGGAAGAACACCACAGACCAGA‐3′.

Reaction conditions were identical to mitochondrial DNA amplification.

- Thermal cycling: 94°C, 8 min → 40 cycles (94°C for 30 s, 58°C for 30 s, 72°C for 20 s) → final extension 72°C, 7 min. Products were visualized on 3% SYBR™ Safe‐stained agarose gels.

### 2.4 p14 protein

**FIGURE 5:**
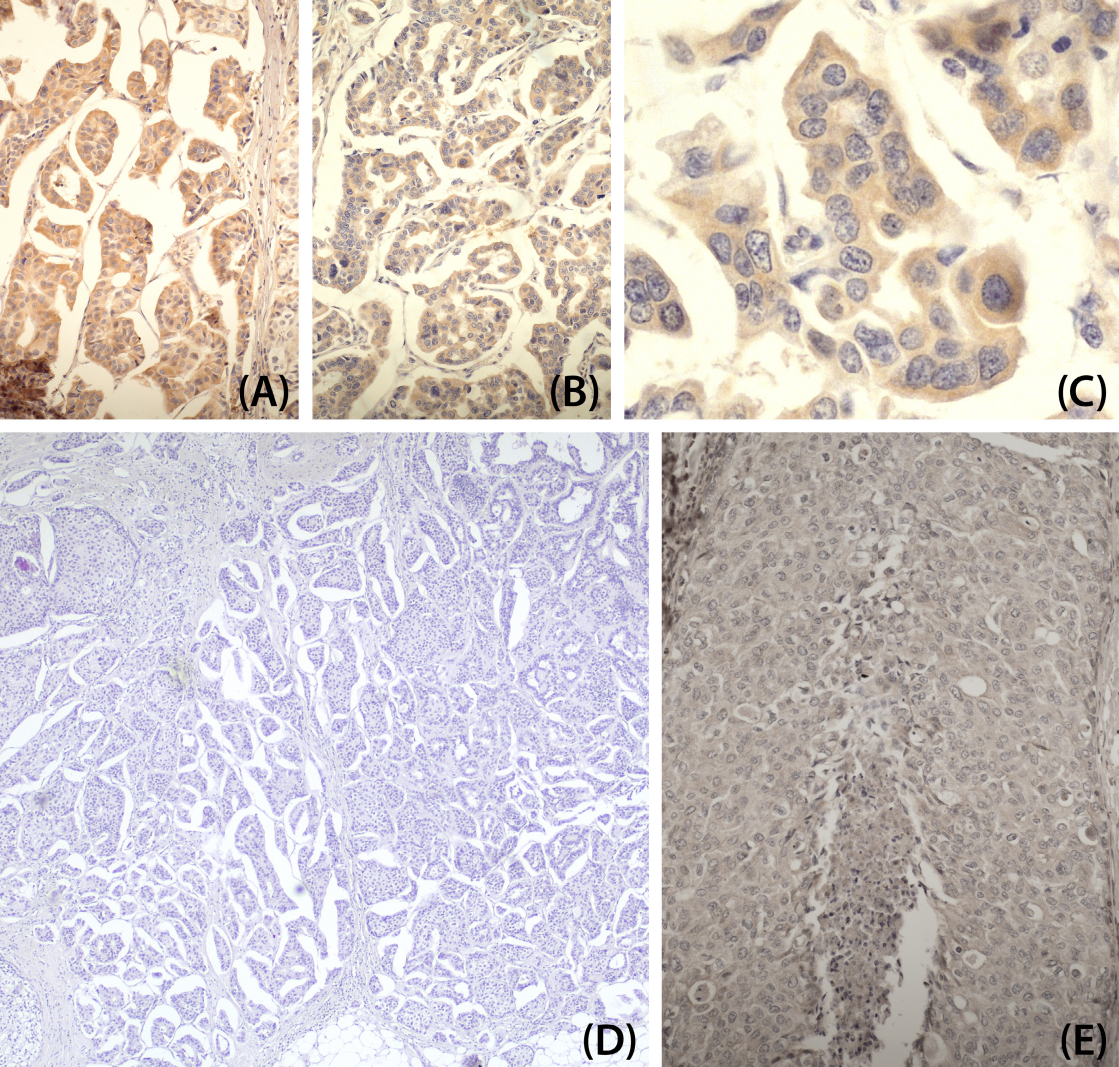
Immunohistochemical analysis of p14 protein. (A–C) Two cases of *ENV*‐positive NST invasive carcinomas showing p14 positivity; C is an enlargement of B. (D) negative control of A, showing the absence of antibody staining. (E) an *ENV*‐negative NST invasive carcinoma, also negative for p14. Positivity is limited to cancer cells.

A subset of invasive tumors was analyzed for the p14 phosphoprotein, the MMTV/HMTV *ENV* signal peptide.^33^ Immunohistochemistry (IHC) was performed using a primary rabbit polyclonal anti‐MMTV‐p14 antibody (1:500 dilution). Staining was developed using diaminobenzidine (DAB) chromogen (DAKO) and counterstained with hematoxylin. Negative controls omitted the primary antibody (Figure 5).

### 2.5 Biological markers

Estrogen and progesterone receptors (ER, PR) and Her2 were routinely evaluated.

### 2.6 Statistical analysis

Descriptive statistics were performed using Microsoft Excel.

## 3 RESULTS

### 3.1 *ENV* sequence

The *ENV* sequence corresponding to GenBank accession number AF243039 was confirmed by Sanger sequencing (Figures 2 and 3). The absence of murine DNA (mitochondrial DNA and IAP LTRs) was verified (Figure 4).

### 3.2 Distribution of the *env* sequence among histotypes

The overall positivity rate for the *ENV* sequence was 20% (Table 1 and Figure 1). NST carcinomas exhibited a 28% positivity rate, with variations across histological grades: G1: 20%, G2: 44%, (double the rate of G1 and G3), and G3: 25%.

Among special type carcinomas, none of the 16 apocrine carcinoma and seven medullary carcinoma cases contained viral sequences; metaplastic carcinoma was predominantly negative, with only one positive case out of 20 (5%). These histotypes are closely associated with hereditary breast cancer syndromes (Tables 1 and 2 and Figure 1).

**TABLE 2:** HBRV in histotypes associated with hereditary cancer syndromes.

Histotype	Total number	No. of positive cases	% of positivity
1. Apocrine	16	0	0%
2. Medullary	7	0	0%
3. Metaplastic	20	1	5%
Total	43	1	2%

*Note*: Medullary and apocrine carcinomas were negative for HBRV, while metaplastic carcinoma was largely negative.

Cribriform and micropapillary carcinomas had a positivity rate <10%, while other histotypes ranged from 15% for mucinous carcinoma to 59% for tubular carcinoma.

Extensive DCIS is defined as DCIS associated with invasive carcinoma, as described in the Materials and Methods section. Extensive DCIS exhibited a 19% positivity rate, comparable to the mean values of pure invasive tumors (22.5%) and combined invasive and extensive DCIS cases (21%). These findings align closely with the 26% positivity rate observed in invasive breast carcinomas across 51 patient groups worldwide, with a total of 5015 patients.^2^

The exceptionally high positivity in tubular carcinoma, the negativity in apocrine and medullary carcinoma, the near‐negativity in metaplastic carcinoma, and the findings for extensive DCIS will be discussed further.

### 3.3 p14 protein

To assess viral protein expression, a subset of 40 invasive tumors underwent IHC analysis for p14 protein, the signal peptide of MMTV *ENV* identified by Jacob Hochman,^33^ who provided specific antibodies for this study. IHC positivity was observed in 24 cases, while *ENV* sequence was positive in 22 cases. Both methods yielded concordant results in 38 cases (95%). Examples of positive cases (NST) and negative controls are shown in Figure 5. Positivity is limited to neoplastic cells.

### 3.4 Biological markers

No correlation was observed between HBRV positivity and biological parameters (ERs, PRs, HER2), consistent with previous studies.^34^

## 4 DISCUSSION

A viral etiology for sporadic breast carcinoma is not unexpected, given that several cancers, including those of the cervix, anogenital region, mouth, throat, nasopharynx, esophagus, stomach, liver, and lymphatic tissue (e.g., Burkitt's lymphoma and adult T‐cell leukemia), as well as Kaposi's sarcoma, have well‐established viral origins. Additionally, mesothelioma and glioblastoma have also been suggested to have viral links.

Over the past 30 years, mounting evidence has supported a viral etiology for both human breast cancer and primary biliary cholangitis. Numerous studies have detected MMTV‐like sequences in BC, with frequencies exceeding the 40% initially reported.^2^

Lower detection rates may reflect geographical differences in virus epidemiology or result from the inclusion of hereditary carcinomas, which have previously been shown to lack an association with HBRV. Additionally, specific histotypes described in this study exhibit little or no HBRV positivity, which may contribute to variability in detection rates. Moreover, viral loss must be considered, as discussed later in relation to the “hit and run” hypothesis.

Viral sequences have been detected in the dental calculus of individuals from the Copper Age^27^ and in the saliva of present‐day individuals.^20^ Furthermore, an MMTV‐like betaretrovirus has been isolated from patients with PBC.^25^ Significantly, experimental studies have demonstrated that MMTV can infect human mammary cells.^22^, ^24^ This growing body of evidence supports the existence of a human betaretrovirus, HBRV, a homolog of MMTV, with saliva emerging as a potential route of transmission.

The identification of MMTV common integration sites (CIS) has been pivotal in human breast cancer research, helping to uncover genes and pathways associated with BC development and carcinogenesis in general.^35^ Consequently, it is plausible that HBRV exerts oncogenic effects through insertional mutagenesis, though the precise molecular mechanisms remain incompletely understood. Notably, HBRV insertion in human BC has been preliminary reported,^25^ supporting its potential role in breast carcinogenesis.

In mice, the randomness of insertional mutagenesis is compensated by the abundant quantity of viral particles in the milk, while in human milk, viral particles have only rarely been identified. HBRV sequences are frequently found in human saliva, though viral particles themselves have yet to be detected.

These findings suggest that alternative mechanisms of transformation may be involved. Brian Salmons and Walter Günzburg^36^, ^37^, ^38^ have carefully discussed this point, emphasizing that several viral proteins have characteristics of an oncogene. Specific references can be found also in a recent review.^2^

- *Env*: the env protein's surface unit (SU) contains an immunoreceptor tyrosine‐based activation motif (ITAM), which can initiate malignant transformation of mammary epithelial cells by suppressing apoptosis through  Src tyrosine kinase signaling. Notably, the Env protein of another betaretrovirus, Jaagsiekte sheep retrovirus (JSRV), plays a role in ovine pulmonary adenocarcinoma (OPA) oncogenesis.
- *Sag*: the superantigen has been shown to stimulate epithelial cell proliferation and promote the tumorigenicity of hyperplastic mammary epithelial cells.
- *Gag*: MMTV gag‐encoded proteins have been demonstrated to directly contribute to the transformation of mammary epithelial cells.
- *Naf*: as a transcriptional repressor of the retroviral genome, it can induce differential expression of proteins and reduce epithelial cell growth.
- *Rem and p14 protein*: the signal peptide (SP) of MMTV, also referred to as the p14 peptide/protein, is derived from the Rem or Env proteins through doubly spliced mRNA. Rem is an internally truncated form of the Env protein. The p14/SP phosphoprotein undergoes phosphorylation at distinct sites, Serine 18 and Serine 65, by PKC and CK2 kinases, respectively. Notably, p14/SP can act as an oncogene when phosphorylated by CK2 or function as a tumor suppressor when phosphorylated by PKC.
- *Activation of a Second Oncogenic Virus*: MMTV might activate other oncogenic viruses, such as EBV and HPV, which have been implicated in human breast cancer. For instance, HIV‐1 Tat protein can activate KSHV (Kaposi's sarcoma‐associated herpesvirus) by regulating PI3K/PTEN/AKT/GSK‐3β pathway.
- *Cell fusion*: viruses can propagate between host cells by inducing the fusion of their cell membranes. This process can result in tetraploidy, which may lead to cancer‐associated aneuploidy through deregulation of p53. Notably, all human oncogenic viruses have the capacity to induce cell fusion and inhibit the functions of p53 and Rb. Virus‐positive premalignant lesions more frequently exhibit tetraploid tumor cells.
- *ERVs/HERVs*: in certain mouse strains, the MMTV genome can integrate into the germline after infection, allowing vertical transmission. This results in the permanent incorporation of the virus into the host genome as an endogenous retrovirus (ERV), known as Mtv. Although ERVs are typically non‐pathogenic, Mtv in specific mouse strains can induce mammary tumors and produce mature viral particles in the milk. Human endogenous retroviruses (HERVs) are the human analog of ERVs. HERV‐K, a betaretrovirus‐like supergroup, shares sequence similarities with MMTV and has been associated with various human cancers, including breast cancer. The potential interaction between exogenous and endogenous MMTV remains an interesting area of investigation. Recently, transcriptomic analyses have proven useful in characterizing HERV expression in human BC. This approach could serve as a powerful tool for exploring the relationship between HBRV and HERVs.^39^

Recent studies suggest additional oncogenic mechanisms, though independent confirmation is still needed:

- *Global down regulation of gene expression*: the expression of MMTV has been shown to negatively affect gene expression, leading to the down‐regulation of critical signaling pathways, including Wnt, Hedgehog, Focal adhesion, Rap1, Hippo, Egfr, Prolactin, PI3K‐Akt–mTOR, Ras, Metabolism, Inflammation, Estrogen, Glutathione, and Vegf. This widespread disruption of gene expression may contribute to cell transformation. Additionally, researchers have identified 12 hub genes, either upregulated or downregulated, implicated in the progression of human BC.^40^
- *miRNA cluster miR‐92*: MMTV has been found to alter the expression of the host miR‐17‐92 cluster, frequently dysregulated in various cancers, particularly BC.^41^

The microscopic architecture of cancer is not a random occurrence; rather, it reflects specific molecular alterations within the tumor. Genetically engineered mouse models (GEMMs) have played a crucial role in clarifying the link between genotype and phenotype in mammary tumors. Key findings include the following^42^, ^43^, ^44^, ^45^, ^46^, ^47^, ^48^, ^49^:

- *K‐ras, H‐ras, ErbB2*, and *Met*: these genes drive the development of tumors exhibiting epithelial‐mesenchymal transition (EMT) morphology, resembling basal‐like tumors in human BC.
- *Myc*: this oncogene can generate both EMT‐like tumors and epithelial tumors with papillary or microacinar architecture.
- *Wap‐Int3 (Notch 4)*: induces tumors with a papillary morphology.
- *Hedgehog and WNT pathways*: associated with squamous features in tumors.
- *Met*: specifically linked to the development of basal‐like neoplasms.
- *E2F1 and E2F2 transcription factors*: loss of E2F1 reduces the incidence of adenosquamous tumors, whereas loss of E2F2 increases it.
- *Wnt1*: capable of transforming progenitor cells of both luminal and myoepithelial lineages, resulting in tumors of diverse histotypes.
- *PyMT* (polyoma middle T): tumor outcomes are promoter dependent: (1) *MMTV‐PyMT* activates signaling pathways involving Src, Ras, and PI3K, leading to adenocarcinomas driven by luminal epithelial cells; (2) *EF1α‐PyMT10C lentivirus* targets both luminal and basal cells, producing tumors with either epithelial or EMT characteristics; (3) *Muc‐PyMT10C lentivirus* (mucin 1 promoter) generates a rare lipid‐rich tumor phenotype.

In contrast, the link between histotypes and their etiological factors remains largely unclear. Squartini's seminal paper^30^ emphasized that the structural and biological characteristics of cancer are directly influenced by the specific strain of the causative MMTV. Notably, in 2006, James Lawson and colleagues proposed a potential association between the presence of MMTV sequences and certain types of human breast carcinoma.^50^

This study explores the distribution of HBRV‐*ENV/*Env gene and protein across different histotypes in a large cohort of human BC cases. Our findings reveal that HBRV is present in all BC histotypes, except medullary, apocrine, and metaplastic carcinomas. Previous research^21^ has shown that hereditary BRCA‐related breast cancers are nearly always negative for viral sequences. This suggests a distinct etiopathogenetic pathway driven mainly by inherited germline genetic alterations rather than external factors. Interestingly, medullary carcinoma^51^ and metaplastic carcinoma^52^ are closely associated with hereditary breast cancer syndromes involving BRCA genes. Notably, apocrine carcinoma is linked to Cowden syndrome,^53^ a component of the PTEN hamartomatous tumor syndrome, which also increases the risk of BC and other malignancies.

As previously discussed, in the murine model, MMTV plays a critical role in initiating the neoplastic process. This occurs through the expression of Int genes, which drive the formation of early hyperplastic preinvasive lesions. The subsequent progression of the disease, including the development of invasive carcinoma and metastatic dissemination, is governed by molecular pathways activated by the same Int genes. These concepts have been extensively discussed in several authoritative studies.^7^, ^9^, ^35^, ^54^, ^55^, ^56^

The “hit and run” hypothesis, first formulated in the 1976^57^ and recently revisited,^58^ suggests that an oncogenic virus can initiate the neoplastic process but may later be lost, leaving the cancer cell population regulated by the molecular pathways it initially activated. These pathways then drive the subsequent phases of cancer progression, invasion and metastasis. This hypothesis implies that the actual contribution of oncogenic viruses to cancer development may be underestimated.

Several findings, some already described in human BC and others newly reported in this study, warrant a unified interpretation in light of the “hit and run” hypothesis:

- *Ductal carcinoma* in situ (*DCIS*), an early preinvasive lesion, exhibits the highest viral positivity rate ever observed, at 82%.^17^
- *Tubular carcinoma* demonstrates the highest HBRV positivity rate among invasive histotypes, approximately 60%. Molecular studies^59^ suggest that this represents a distinct subtype of invasive cancer, arrested in the early stages of progression. Its malignancy is typically limited to local invasion without advancing to a more aggressive form, making it biologically more comparable to DCIS than to invasive tumors. Notably, tubular carcinoma frequently coexists with DCIS foci.
- *Invasive sporadic carcinoma* exhibits a significantly lower mean positivity rate, reduced by at least half^2^, ^17^ compared to DCIS. Moreover, quantitative PCR analysis has demonstrated a reduction or loss of the virus in the invasive stage compared to DCIS.^17^
- *Extensive DCIS*, which constitutes an in situ component within an invasive carcinoma, displays an HBRV positivity rate of 19%, comparable to that of pure NST (no special type) invasive cancers.
- *Biological markers*, including *estrogen* and *progesterone receptor expression* and *HER2 amplification*, do not correlate with HBRV positivity.

These data can be read as follows: (1) In DCIS and in tubular carcinoma, where invasive and metastatic molecular pathways have not yet been activated, the virus remains present and actively replicates. (2) In other invasive histotypes, including extensive DCIS, tumor progression is driven by pathways activated by Int genes, rendering the virus unnecessary. Consequently, it may be lost. Expanding invasive cell clones, which become virus‐free or harbor a low viral load, outcompete still‐infected clones. (3) Since biological markers reflect the characteristics of the invasive cell population, which is no longer connected to the virus, they cannot be correlated with the viral presence, which is diminished or absent.

At the same time, this data add strength to the “hit and run” hypothesis.

The precise mechanisms underlying the “hit and run” hypothesis remain incompletely understood. Potential contributing factors include viral loss due to genomic or chromosomal rearrangements, immune‐mediated clearances, or as‐yet unidentified processes.

The overall findings presented in this article align with previous reports suggesting that the virus plays a role in the etiology of nearly all sporadic breast carcinoma cases. This is supported by the 82% HBRV positivity rate in pure DCIS cases,^17^ the 80% positivity rate in invasive BC documented by Axel et al. in 1972,^13^ the detection of the virus across all sporadic carcinoma histotypes, and, finally, the possibility of viral loss. Insights from the murine model and GEMMs suggest that the diverse histotypes observed in human BC may arise from the activation of distinct molecular pathways by Int genes or from alternative oncogenic mechanisms unrelated to insertional mutagenesis. Additionally, the presence of different HBRV strains remains a possibility, adding further complexity to the etiopathogenesis of breast cancer.

Some studies have proposed that the viral sequences detected in human specimens may originate from human endogenous retroviruses (HERV). However, the sequences identified in human tissues have been conclusively demonstrated to be of exogenous origin, distinct from both other animal exogenous betaretroviruses and all HERV‐K HMLs.^14^, ^27^ Regarding the potential contamination of human tissues with murine materials: (a) the presence of HBRV in human breast cancer has been consistently observed worldwide, with most studies utilizing FFPE blocks sourced from pathology department archives; (b) most laboratories involved in these studies did not house mice; (c) this study, like others, shows the concurrent presence of the *ENV* gene and the p14 protein; and (d) it is implausible that contamination could account for the significant differences in HBRV positivity between sporadic and hereditary tumors, as demonstrated in prior research and corroborated by the present study, given that both groups of tissues were obtained from the same archives.^2^ Notably, phylogenetic analysis has revealed a significant relationship between human viral sequences and HMTV, MMTV, and MMTV‐C3H. Negative critiques often rely on outdated studies, some published as long as 20 years ago,^60^ whose conclusions have since been invalidated by more recent and rigorous investigations. These concerns have been comprehensively addressed in recent publications,^1^, ^2^ which provide further clarity and reinforce the reliability of the experimental methods employed. Ultimately, the conclusive identification of HBRV insertion in human BC would contribute to resolving this controversy.^25^

## 5 CONCLUSION

This study suggests that the Human Betaretrovirus plays a role in the etiology of nearly all cases of human sporadic breast carcinoma, being detected across all histotypes except those typically associated with hereditary breast carcinoma, where the absence of a viral agent is well established. Based on the murine MMTV model, the diversity of histological subtypes observed in human sporadic breast carcinoma may result from the activation of different molecular pathways, as occurs during insertional mutagenesis, from alternative oncogenic mechanisms unrelated to insertional mutagenesis, or from the potential existence of diverse HBRV strains.

## AUTHOR CONTRIBUTIONS

**Prospero Civita:** Investigation; methodology; validation; formal analysis; writing – review and editing. **Chiara Maria Mazzanti:** Supervision; investigation; methodology; validation; formal analysis; writing – review and editing. **Francesca Lessi:** Investigation; methodology; validation; formal analysis; writing – review and editing. **Caterina Marchiò:** Investigation; validation; writing – review and editing. **Cristian Scatena:** Investigation; validation; writing – review and editing. **Michele Menicagli:** Investigation; methodology; validation; writing – review and editing. **Matteo Ghilli:** Investigation; validation; writing – review and editing. **Manuela Roncella:** Investigation; validation; writing – review and editing. **Antonio Giuseppe Naccarato:** Investigation; validation; writing – review and editing. **Anna Sapino:** Investigation; validation; writing – review and editing. **Jacob Hochman:** Investigation; validation; writing – review and editing. **Mauro Pistello:** Investigation; validation; writing – review and editing. **Generoso Bevilacqua:** Conceptualization; writing – original draft; formal analysis; project administration; supervision.

## FUNDING INFORMATION

Ministero della Salute ‐ Italian Ministry of Health. Next Generation EU—PNRR M6C2—Investimento 2.1 Valorizzazione e potenziamento della ricerca biomedica del SSN (Codice progetto PNRR‐MAD‐2022‐12376570, CAP: PNRR0308, CDC: 03080103, UDP: 0308PNRR—FONDI NEXTGENERATIONEU).

## CONFLICT OF INTEREST STATEMENT

The authors declare no conflict of interests.

## ETHICS STATEMENT

Specimens were collected anonymously from the archives according to the recommendations of the Ethics Committee of the University of Pisa.

## Data Availability

The data that support the findings of this study are available from the corresponding author upon reasonable request.

## References

[1] Lawson JS , Glenn WK . The viral origins of breast cancer. Infect Agent Cancer. 2024;19(1):39. doi:10.1186/s13027-024-00595-2 39187871 PMC11346025

[2] Bevilacqua G . The viral origin of human breast cancer: from the Mouse Mammary Tumor Virus (MMTV) to the Human Betaretrovirus (HBRV). Viruses. 2022;14(8):1704. doi:10.3390/v14081704 36016325 PMC9412291

[3] Endogenous Hormones and Breast Cancer Collaborative Group . Circulating sex hormones and breast cancer risk factors in postmenopausal women: reanalysis of 13 studies. Br J Cancer. 2011;105(5):709‐722. doi:10.1038/bjc.2011.254 21772329 PMC3188939

[4] Bhardwaj P , Au CC , Benito‐Martin A , et al. Estrogens and breast cancer: mechanisms involved in obesity‐related development, growth and progression. J Steroid Biochem Mol Biol. 2019;189:161‐170. doi:10.1016/j.jsbmb.2019.03.002 30851382 PMC6502693

[5] Tin Tin S , Smith‐Byrne K , Ferrari P , et al. Alcohol intake and endogenous sex hormones in women: meta‐analysis of cohort studies and Mendelian randomization. Cancer. 2024;130(19):3375‐3386. doi:10.1002/cncr.35391 38824654

[6] Loroña NC , Othus M , Malone KE , Linden HM , Tang MC , Li CI . Alcohol, smoking, and risks of breast cancer recurrence and mortality among women with luminal, triple‐negative, and HER2‐overexpressing breast cancer. Cancer Epidemiol Biomarkers Prev. 2024;33(2):288‐297. doi:10.1158/1055-9965 38019269 PMC10872526

[7] Cardiff RD , Kenney N . Mouse mammary tumor biology: a short history. Adv Cancer Res. 2007;98:53‐116. doi:10.1016/S0065-230X(06)98003-8 17433908

[8] Faschinger A , Rouault F , Sollner J , et al. Mouse Mammary Tumor Virus integration site selection in human and mouse genomes. J Virol. 2008;82(3):1360‐1367. doi:10.1128/JVI.02098-07 18032509 PMC2224419

[9] Dudley JP , Golovkina TV , Ross SR . Lessons learned from Mouse Mammary Tumor Virus in animal models. ILAR J. 2016;57(1):12‐23. doi:10.1093/ilar/ilv044 27034391 PMC5007637

[10] Ball JK , Diggelmann H , Dekaban GA , et al. Alterations in the U3 region of the long terminal repeat of an infectious thymotropic type B retrovirus. J Virol. 1988;62(8):2985‐2993. doi:10.1128/JVI.62.8.2985-2993.1988 2839715 PMC253737

[11] Yanagawa S , Kakimi K , Tanaka H , et al. Mouse Mammary Tumor Virus with rearranged long terminal repeats causes murine lymphomas. J Virol. 1993;67(1):112‐118. doi:10.1128/JVI.67.1.112-118.1993 7677952 PMC237343

[12] Bentvelzen P . Host‐virus interactions in murine mammary carcinogenesis. Biochim Biophys Acta. 1974;355(3–4):236‐259. doi:10.1016/0304-419x(74)90012-2 4142395

[13] Axel R , Gulati SC , Spiegelman S . Particles containing RNA‐instructed DNA polymerase and virus‐related RNA in human breast cancers. Proc Natl Acad Sci U S A. 1972;69(11):3133‐3137. doi:10.1073/pnas.69.11.3133 4117770 PMC389720

[14] Wang Y , Holland JF , Bleiweiss IJ , et al. Detection of Mammary Tumor Virus env gene‐like sequences in human breast cancer. Cancer Res. 1995;55(22):5173‐5179. 7585568

[15] Liu B , Wang Y , Melana SM , et al. Identification of a proviral structure in human breast cancer. Cancer Res. 2001;61(4):1754‐1759. 11245493

[16] Zammarchi F , Pistello M , Piersigilli A , et al. MMTV‐like sequences in human breast cancer: a fluorescent PCR/laser microdissection approach. J Pathol. 2006;209(4):436‐444. doi:10.1002/path.1997 16710841

[17] Mazzanti CM , Al Hamad M , Fanelli G , et al. A Mouse Mammary Tumor Virus env‐like exogenous sequence is strictly related to progression of human sporadic breast carcinoma. Am J Pathol. 2011;179(4):2083‐2090. doi:10.1016/j.ajpath.2011.06.046 21854742 PMC3181336

[18] Nartey T , Mazzanti CM , Melana S , et al. Mouse Mammary Tumor‐like Virus (MMTV) is present in human breast tissue before development of virally associated breast cancer. Infect Agent Cancer. 2017;12:1. doi:10.1186/s13027-016-0113-6 28053656 PMC5209856

[19] Johal H , Faedo M , Faltas J , et al. DNA of Mouse Mammary Tumor Virus‐like virus is present in human tumors influenced by hormones. J Med Virol. 2010;82(6):1044‐1050. doi:10.1002/jmv.21754 20419820

[20] Mazzanti CM , Lessi F , Armogida I , et al. Human saliva as route of inter‐human infection for Mouse Mammary Tumor Virus. Oncotarget. 2015;6(21):18355‐18363. doi:10.18632/oncotarget.4567 26214095 PMC4621895

[21] Naccarato AG , Lessi F , Zavaglia K , et al. Mouse Mammary Tumor Virus (MMTV)‐like exogenous sequences are associated with sporadic but not hereditary human breast carcinoma. Aging (Albany NY). 2019;11(17):7236‐7241. doi:10.18632/aging.102252 31518337 PMC6756874

[22] Indik S , Günzburg WH , Kulich P , Salmons B , Rouault F . Rapid spread of Mouse Mammary Tumor Virus in cultured human breast cells. Retrovirology. 2007;4:73. doi:10.1186/1742-4690-4-73 17931409 PMC2169256

[23] Melana SM , Nepomnaschy I , Sakalian M , et al. Characterization of viral particles isolated from primary cultures of human breast cancer cells. Cancer Res. 2007;67(18):8960‐8965. doi:10.1158/0008-5472.CAN-06-3892 17875739

[24] Indik S , Günzburg WH , Salmons B , Rouault F . Mouse Mammary Tumor Virus infects human cells. Cancer Res. 2005;65(15):6651‐6659. doi:10.1158/0008-5472.CAN-04-2609 16061645

[25] Zadian S , Verma V , Ford G , et al. Demonstration of human betaretrovirus infection in patients with breast cancer by identifying cellular immune responses and proviral integrations. 28th West Coast Retrovirus Meeting, Palm Springs, CA, USA, 3–5 October 2024, abstract book p. 20. 2024.

[26] Goubran M , Wang W , Indik S , et al. Isolation of a human Betaretrovirus from patients with primary biliary cholangitis. Viruses. 2022;14(5):886. doi:10.3390/v14050886 35632628 PMC9146342

[27] Lessi F , Grandi N , Mazzanti CM , et al. A human MMTV‐like betaretrovirus linked to breast cancer has been present in humans at least since the copper age. Aging (Albany NY). 2020;12(16):15978‐15994. doi:10.18632/aging.103780 32735554 PMC7485742

[28] Xu L , Sakalian M , Shen Z , Loss G , Neuberger J , Mason A . Cloning the human betaretrovirus proviral genome from patients with primary biliary cirrhosis. Hepatology. 2004;39(1):151‐156. doi:10.1002/hep.20024 14752833

[29] Dunn TB . Morphology of Mammary Tumors in Mice. In: Homburger F , ed. Paul B Hoeber Inc; 1958:38‐84.

[30] Squartini F , Rossi G , Paoletti I . Characters of mammary tumours in BALB/c female mice foster‐nursed by C3H and RIII mothers. Nature. 1963;197:505‐506. doi:10.1038/197505a0 13978585

[31] Katz E , Lareef MH , Rassa JC , et al. MMTV Env encodes an ITAM responsible for transformation of mammary epithelial cells in three‐dimensional culture. J Exp Med. 2005;201(3):431‐439. doi:10.1084/jem.20041471 15684322 PMC2213037

[32] Robinson MJ , Erlwein OW , Kaye S , et al. Mouse DNA contamination in human tissue tested for XMRV. Retrovirology. 2010;7:108. doi:10.1186/1742-4690-7-108 21171966 PMC3019155

[33] Hochman J , Braitbard O . Life after cleavage: the story of a β‐retroviral (MMTV) signal peptide‐from murine lymphoma to human breast cancer. Viruses. 2022;14(11):2435. doi:10.3390/v14112435 36366533 PMC9694287

[34] de Sousa Pereira N , Akelinghton Freire Vitiello G , Karina Banin‐Hirata B , et al. Mouse Mammary Tumor Virus (MMTV)‐like *env* sequence in Brazilian breast cancer samples: implications in clinicopathological parameters in molecular subtypes. Int J Environ Res Public Health. 2020;17(24):9496. doi:10.3390/ijerph17249496 33352945 PMC7766913

[35] Callahan R , Mudunur U , Bargo S , et al. Genes affected by Mouse Mammary Tumor Virus (MMTV) proviral insertions in mouse mammary tumors are deregulated or mutated in primary human mammary tumors. Oncotarget. 2012;3(11):1320‐1334. doi:10.18632/oncotarget.682 23131872 PMC3717796

[36] Salmons B , Günzburg WH . Revisiting a role for a mammary tumor retrovirus in human breast cancer. Int J Cancer. 2013;133(7):1530‐1535. doi:10.1002/ijc.28210 23580334

[37] Salmons B , Lawson JS , Günzburg WH . Recent developments linking retroviruses to human breast cancer: infectious agent, enemy within or both? J Gen Virol. 2014;95(Pt 12):2589‐2593. doi:10.1099/vir.0.070631-0 25217613

[38] Salmons B , Günzburg WH . Tumorigenesis mechanisms of a putative human breast cancer retrovirus. Austin Virol Retrovirol. 2015;2:1010.

[39] Liang B , Yan T , Wei H , et al. HERVK‐mediated regulation of neighboring genes: implications for breast cancer prognosis. Retrovirology. 2024;21(1):4. doi:10.1186/s12977-024-00636-z 38388382 PMC10885364

[40] Ahmad W , Panicker NG , Akhlaq S , et al. Global down‐regulation of gene expression induced by Mouse Mammary Tumor Virus (MMTV) in normal mammary epithelial cells. Viruses. 2023;15(5):1110. doi:10.3390/v15051110 37243196 PMC10223558

[41] Baby J , Gull B , Ahmad W , et al. The host miR‐17‐92 cluster negatively regulates Mouse Mammary Tumor Virus (MMTV) replication primarily via cluster member miR‐92a. J Mol Biol. 2024;436(20):168738. doi:10.1016/j.jmb.2024.168738 39117177

[42] Hollern DP , Swiatnicki MR , Andrechek ER . Histological subtypes of mouse mammary tumors reveal conserved relationships to human cancers. PLoS Genet. 2018;14(1):e1007135. doi:10.1371/journal.pgen.1007135 29346386 PMC5773092

[43] Holloway KR , Sinha VC , Bu W , et al. Targeting oncogenes into a defined subset of mammary cells demonstrates that the initiating oncogenic mutation defines the resulting tumor phenotype. Int J Biol Sci. 2016;12(4):381‐388. doi:10.7150/ijbs.12947 27019623 PMC4807158

[44] Hollern DP , Honeysett J , Cardiff RD , Andrechek ER . The E2F transcription factors regulate tumor development and metastasis in a mouse model of metastatic breast cancer. Mol Cell Biol. 2014;34(17):3229‐3243. doi:10.1128/MCB.00737-14 24934442 PMC4135561

[45] Smith BA , Shelton DN , Kieffer C , et al. Targeting the PyMT oncogene to diverse mammary cell populations enhances tumor heterogeneity and generates rare breast cancer subtypes. Genes Cancer. 2012;3(9–10):550‐563. doi:10.1177/1947601913475359 23486760 PMC3591097

[46] Andrechek ER , Cardiff RD , Chang JT , et al. Genetic heterogeneity of Myc‐induced mammary tumors reflecting diverse phenotypes including metastatic potential. Proc Natl Acad Sci U S A. 2009;106(38):16387‐16392. doi:10.1073/pnas.0901250106 19805309 PMC2752567

[47] Ponzo MG , Lesurf R , Petkiewicz S , et al. Met induces mammary tumors with diverse histologies and is associated with poor outcome and human basal breast cancer. Proc Natl Acad Sci U S A. 2009;106(31):12903‐12908. doi:10.1073/pnas.0810402106 19617568 PMC2722321

[48] Li Y , Welm B , Podsypanina K , et al. Evidence that transgenes encoding components of the Wnt signaling pathway preferentially induce mammary cancers from progenitor cells. Proc Natl Acad Sci U S A. 2003;100(26):15853‐15858. doi:10.1073/pnas.2136825100 14668450 PMC307657

[49] Rosner A , Miyoshi K , Landesman‐Bollag E , et al. Pathway pathology: histological differences between ErbB/Ras and Wnt pathway transgenic mammary tumors. Am J Pathol. 2002;161(3):1087‐1097. doi:10.1016/S0002-9440(10)64269-1 12213737 PMC1867237

[50] Lawson JS , Tran DD , Carpenter E , et al. Presence of mouse mammary tumour‐like virus gene sequences may be associated with morphology of specific human breast cancer. J Clin Pathol. 2006;59(12):1287‐1292. doi:10.1136/jcp.2005.035907 16698952 PMC1860546

[51] Wittersheim M , Büttner R , Markiefka B . Genotype/phenotype correlations in patients with hereditary breast cancer. Breast Care (Basel). 2015;10(1):22‐26. doi:10.1159/000380900 25960721 PMC4395815

[52] Corso G , Marabelli M , Calvello M , et al. Germline pathogenic variants in metaplastic breast cancer patients and the emerging role of the BRCA1 gene. Eur J Hum Genet. 2023;31(11):1275‐1282. doi:10.1038/s41431-023-01429-2 37460658 PMC10620155

[53] Banneau G , Guedj M , MacGrogan G , et al. Molecular apocrine differentiation is a common feature of breast cancer in patients with germline PTEN mutations. Breast Cancer Res. 2010;12(4):R63. doi:10.1186/bcr2626 20712882 PMC2949656

[54] Nusse R . The int genes in mammary tumorigenesis and in normal development. Trends Genet. 1988;4(10):291‐295. doi:10.1016/0168-9525(88)90172-2 3076290

[55] Tsukamoto AS , Grosschedl R , Guzman RC , Parslow T , Varmus HE . Expression of the int‐1 gene in transgenic mice is associated with mammary gland hyperplasia and adenocarcinomas in male and female mice. Cell. 1988;55(4):619‐625. doi:10.1016/0092-8674(88)90220-6 3180222

[56] Callahan R , Smith GH . Common integration sites for MMTV in viral induced mouse mammary tumors. J Mammary Gland Biol Neoplasia. 2008;13(3):309‐321. doi:10.1007/s10911-008-9092-6 18709449 PMC3104473

[57] Skinner GR . Transformation of primary hamster embryo fibroblasts by type 2 simplex virus: evidence for a “hit and run” mechanism. Br J Exp Pathol. 1976;57(4):361‐376. 183803 PMC2041152

[58] Ferreira DA , Tayyar Y , Idris A , McMillan NAJ . A “hit‐and‐run” affair: a possible link for cancer progression in virally driven cancers. Biochim Biophys Acta Rev Cancer. 2021;1875(1):188476. doi:10.1016/j.bbcan.2020.188476 33186643

[59] Waldman FM , Hwang ES , Etzell J , et al. Genomic alterations in tubular breast carcinomas. Hum Pathol. 2001;32(2):222‐226. doi:10.1053/hupa.2001.21564 11230710

[60] Mant C , Cason J . A human murine mammary tumour virus‐like agent is an unconvincing aetiological agent for human breast cancer. Rev Med Virol. 2004;14(3):169‐177. doi:10.1002/rmv.427 15124233

